# Hepatobiliary Nontuberculous Mycobacterial Infection Mimicking Malignancy in a Patient with Anti-Interferon-γ Autoantibodies

**DOI:** 10.3390/diagnostics16121774

**Published:** 2026-06-09

**Authors:** Mengmeng Zhang, Qiang Wang, Xi Wu, Dong Wu, Aiming Yang

**Affiliations:** Department of Gastroenterology, Peking Union Medical College Hospital, Chinese Academy of Medical Sciences and Peking Union Medical College, No. 1 Shuaifuyuan, Dongcheng District, Beijing 100730, China

**Keywords:** obstructive jaundice, endoscopic retrograde cholangiopancreatography, hemobilia, Anti-IFN-γ autoantibody, adult-onset immunodeficiency, nontuberculous mycobacteria

## Abstract

Obstructive jaundice is a common digestive disorder with multiple etiologies. Non-tuberculous mycobacterial (NTM) infection is an opportunistic disease that may present with localized pulmonary involvement or disseminated multi-organ manifestations. However, biliary involvement in disseminated NTM infection is rare, and its characteristics and progression remain poorly understood. We report a patient with progressive jaundice who was eventually considered to have probable biliary NTM infection after comprehensive evaluation, exclusion of alternative etiologies, and a favorable therapeutic response despite negative microbiological testing. Endoscopic retrograde cholangiopancreatography (ERCP) was performed to relieve biliary obstruction, but the patient developed recurrent and refractory ampullary bleeding requiring repeated endoscopic interventions. Clinical improvement was achieved following combined antimycobacterial therapy and immunomodulatory treatment. Biliary NTM infection is a rare cause of obstructive jaundice, and ERCP remains necessary for biliary decompression, while post-ERCP bleeding risk should be carefully monitored.

**Figure 1:**
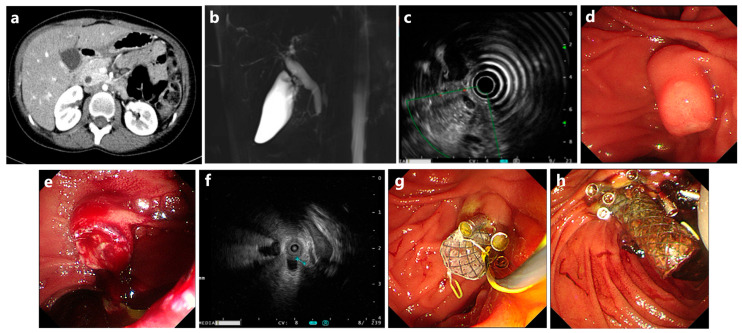
Radiographic and endoscopic images of endoscopic retrograde cholangiopancreatography (ERCP). Adult-onset immunodeficiency caused by anti-IFN-γ autoantibodies is an acquired immunodeficiency syndrome characterized by impaired IFN-γ signaling and susceptibility to disseminated opportunistic infections. A 21-year-old woman was admitted with a 1-year history of progressive jaundice. She was diagnosed with nontuberculous mycobacterial (NTM) infection 8 years ago, presented with fever, multiple enlarged lymphadenopathies, and a culture-positive ulcerated breast nodule for *Mycobacterium avium*. After 1-year anti-mycobacterial treatment (clarithromycin + minocycline→clarithromycin + rifamycin + amikacin→linezolid + levofloxacin, due to abnormal liver function and peripheral neuropathy), ulcerated nodule healed, whereas serum alkaline phosphatase (594 U/L, normal range 35–100) and gamma-glutamyl transpeptidase (204 U/L, normal range 7–45) gradually increased and developed jaundice with progressively increased serum total bilirubin (129.5 μmol/L, normal range 5.1–22.2) and direct bilirubin (105.5 μmol/L, normal range ≤ 6.8). The tests for white blood cell, hemoglobin, platelet, coagulation function (prothrombin time, activated partial thromboplastin time), serum amylase and lipase, serum tumor markers (AFP, CEA), serum protein electrophoresis, serum immunofixation electrophoresis, angiotensin converting enzyme, autoimmune markers (ANA, ANCA, autoimmune hepatitis related antibody, serum IgG, IgA, IgM, IgG4, complement 3 and 4, antiphospholipid syndrome related antibody), liver-related viruses (hepatitis A, B, C, D, E, cytomegalovirus, Epstein–Barr virus, B19), blood aerobic and anaerobic culture, cryptococcal antigen, TORCH-IgM, Brucella serum agglutination test, 1,3-beta-D-glucan test, Galactomannan test were all negative. Anti-interferon (IFN)-γ autoantibodies were positive at a dilution of 1:130,000. Mycobacterial cultures of spectrum, peripheral blood, bone marrow, and metagenomic next-generation sequencing (mNGS) of peripheral blood were also negative. Panel (**a**) enhanced CT and (**b**) magnetic resonance cholangiopancreatography revealed multiple enlarged peri-portal and peritoneal lymph nodes, intra- and extra-hepatic biliary dilatation, and a suspicious filling defect in the distal common bile duct (CBD). Panel (**c**) showed a 1.2 cm hypoechoic ampullary mass involving the distal CBD without acoustic shadowing. Panel (**d**) endoscopic retrograde cholangiopancreatography (ERCP) showed an abnormal bulging papilla resembling an ampullary tumor. Due to a pendulous papilla with bleeding tendency and difficult biliary cannulation, pre-cut sphincterotomy was performed (0.6 cm), followed by biliary biopsy and biliary (6 cm 8.5 Fr) and pancreatic (5 cm 5 Fr) duct stenting. Histopathology demonstrated biliary inflammatory infiltration with negative acid-fast staining. The bile mycobacterial culture and mNGS test were negative. The following day, hematemesis occurred with severe epigastric pain, rapid decreased hemoglobin (hemoglobin 118→88 g/L), and markedly elevated serum amylase (1335 U/L, normal range 35~135) and lipase (2368 U/L, normal range 2~53). Panel (**e**) second ERCP showed that the biliary and pancreatic stents had fallen off and there was active ampullary bleeding, which was closed with titanium clips. Due to elevated total bilirubin (150 μmol/L to 252 μmol/L), panel (**f**) the third ERCP showed 1.5–2.0 cm distal biliary stricture with wall thickening. Panel (**g**) showed that, with intraductal ultrasonography, endobiliary bipolar radiofrequency ablation (10 W for 90 s, 8 W for 60 s) was performed, and a 6 cm 10 Fr biliary covered metal stent was deployed. But the next day, she went into hemorrhagic shock (hemoglobin 90→59 g/L). Panel (**h**) emergency duodenoscope showed ampullary bleeding again, subsequently treated with argon plasma coagulation and titanium clips.

**Figure 2:**
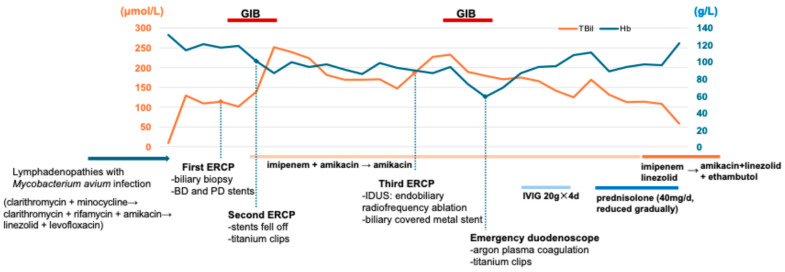
The clinical course of the patient. (ERCP: endoscopic retrograde cholangiopancreatography; IDUS: intraductal ultrasonography; GIB: gastrointestinal bleeding; IVIG: intravenous immunoglobulin). As shown in Figure 2, the entire clinical timeline and process were summarized. Considering unsatisfied bilirubin decrease (total/direct bilirubin 170/135 μmol/L), intravenous immunoglobulin (0.43 mg/kg·d for 4 days) was administered with no significant bilirubin reduction. Then, prednisolone (40 mg/day, 0.86 mg/kg·d) was administered after balancing the risks of uncontrolled inflammation and progressive biliary obstruction against the potential risk of infection exacerbation. Considering she had previously used minocycline and clarithromycin with suboptimal efficacy, rifampin had been discontinued due to peripheral neuropathy. Since she was undergoing severe liver injury, intravenous imipenem and linezolid were initially selected. And after 1 week, the bilirubin obviously decreased (total/direct bilirubin 110/90 μmol/L). She continued prednisolone (20 mg/day), imipenem (0.5 g every 8 h), and intravenous linezolid (0.6 g/day) treatment for 2 weeks, and then prednisolone was tapered by 2.5 mg every 2 weeks. The anti-mycobacterial regimen was adjusted to amikacin (0.4 g/day), oral linezolid (0.6 g/day), and ethambutol (0.75 g/day). Two months later, gastroscopy revealed that the biliary stent had already fallen off, but considering the patient’s bilirubin levels continued to decrease (total/direct bilirubin 52/35 μmol/L), no further endoscopic intervention was performed. During a 1-year follow-up, the patient remains asymptomatic with persistently normal liver function. Adult-onset immunodeficiency due to anti-IFN-γ autoantibody is an emerging acquired syndrome, characterized by severe disseminated opportunistic infections, particularly NTM infection, in HIV-negative patients [1]. Lymph nodes (60%) and skin (41%) are the most commonly involved sites, while hepatobiliary involvement is uncommon (10–20%) [2,3]. In this case, the mass-like change in the ampulla, negative mycobacterial cultures, and recurrent refractory hemobilia after ERCP made the diagnosis and therapy more challenging. Malignancy (ampullary carcinoma, cholangiocarcinoma, lymphoma, etc.) was low probable as the biopsy was negative and no progression occurred during 1-year follow-up. Primary sclerosing cholangitis was unlikely because imaging did not demonstrate multifocal or diffuse intrahepatic biliary strictures. In addition, serum IgG4 and angiotensin-converting enzyme levels were negative, and histopathological findings did not support IgG4-related disease or sarcoidosis. Peripheral blood cultures, fungal test, detectable viral test, as well as peripheral blood and tissue mNGS, did not reveal evidence of other infectious diseases. Furthermore, drug-induced liver injury was also considered unlikely as the patient developed liver injury approximately six months after discontinuation of previous antimicrobial medications, without any history of exposure to other suspicious agents during that period, and her pattern of liver function abnormality was atypical. Although repeated bile culture, histology, and mNGS failed to confirm biliary NTM infection microbiologically, which is one limitation of this case, biliary NTM infection was clinically presumed and supported based on her previous NTM infection history, comprehensive exclusion of other causes, and favorable therapeutic responses to antimycobacterial drugs. Treatments for anti-IFN-γ autoantibody-related immunodeficiency mainly target opportunistic infection and immune dysregulation. Anti-CD20 (rituximab) can eliminate anti-IFN-γ autoantibodies by targeting B cells, which can inhibit IFN-γ–induced pSTAT-1 production and achieve remission of infection [4]. As the patient achieved symptom relief after immunosuppressive and anti-mycobacterial therapy, rituximab was not attempted in this case. The efficacy of corticosteroids, immunoglobulin, and anti-IFN-γ autoantibodies targeted agents has not been ensured. The observed clinical improvement was likely multifactorial, related to biliary decompression, antimycobacterial therapy, and immunomodulation. We predict that immunomodulatory therapy was considered potentially beneficial as anti-IFN-γ autoantibody-associated immunodeficiency involves immune dysregulation and exaggerated inflammatory responses. And it is essential to evaluate the balance of steroid benefits and infection risk in clinical practice. Biliary stricture and progressive jaundice due to NTM infection are indications for ERCP. In this case, recurrent and refractory post-ERCP bleeding and stent migration made the treatment more difficult. The risk of hemobilia depends on the degree of invasiveness of procedures during ERCP, including pre-cut sphincterotomy, inflamed papillary tissue, stent migration or repeat interventions, and patient-dependent variables, including coagulopathy and hypervascular lesions [5,6]. The association between biliary NTM infection and hemobilia remains unknown. The most clinically common cause of infectious hemobilia is ‘tropical hemobilia’, because of parasitic infection [6]. Although it has been speculated in pulmonary NTM infection that a strong immune response or high serum antibody titer against *Mycobacterium avium* may be associated with an increased bleeding tendency [7,8], there is currently no direct evidence in biliary NTM infection, which requires further investigation. The treatment of hemobilia consists of both achieving hemostasis and maintaining bile drainage [6]. Biliary stents can provide a tamponade effect on the bleeding sites and maintain luminal patency of the bile duct [9], and if stent SEMS hemostasis fails, angioembolization can be a rescue therapy [10]. Endobiliary radiofrequency ablation was sometimes performed for refractory benign biliary diseases [11,12], which can be applied combined with biliary stents for biliary stricture and hemobilia [9]. Although this patient was suspected of having a benign infectious lesion, recurrent biliary bleeding and biliary stricture with stent migration occurred, so we tried ablation and metal biliary stent placement as reported by Linz CM et al. [9], but with unsatisfactory effect. In summary, in patients with anti-IFN-γ autoantibody-associated immunodeficiency, biliary NTM infection should be considered as a rare cause of obstructive jaundice. When pathological or microbiological diagnoses are difficult, clinical history, exclusion of alternative etiologies, and therapeutic response may support the diagnosis. ERCP remains important for biliary decompression, while potential post-ERCP hemobilia should be closely monitored, particularly in patients with severe inflammatory biliary lesions. Fully covered metallic stents or endobiliary radiofrequency ablation may be a possible option for refractory hemobilia.

## Data Availability

The data underlying this study are available from the corresponding author upon reasonable request.

## References

[1] Browne S.K., Burbelo P.D., Chetchotisakd P., Suputtamongkol Y., Kiertiburanakul S., Shaw P.A., Kirk J.L., Jutivorakool K., Zaman R., Ding L. (2012). Adult-onset immunodeficiency in Thailand and Taiwan. N. Engl. J. Med..

[2] Hong G.H., Ortega-Villa A.M., Hunsberger S., Chetchotisakd P., Anunnatsiri S., Mootsikapun P., Rosen L.B., Zerbe C.S., Holland S.M. (2020). Natural History and Evolution of Anti-Interferon-γ Autoantibody-Associated Immunodeficiency Syndrome in Thailand and the United States. Clin. Infect. Dis..

[3] Namkoong H., Asakura T., Ishii M., Yoda S., Masaki K., Sakagami T., Iwasaki E., Yamagishi Y., Kanai T., Betsuyaku T. (2019). First report of hepatobiliary Mycobacterium avium infection developing obstructive jaundice in a patient with neutralizing anti-interferon-gamma autoantibodies. New Microbes New Infect..

[4] Browne S.K., Zaman R., Sampaio E.P., Jutivorakool K., Rosen L.B., Ding L., Pancholi M.J., Yang L.M., Priel D.L., Uzel G. (2012). Anti-CD20 (rituximab) therapy for anti-IFN-γ autoantibody-associated nontuberculous mycobacterial infection. Blood.

[5] Rivas A., Pherwani S., Mohamed R., Smith Z.L., Elmunzer B.J., Forbes N. (2023). ERCP-related adverse events: Incidence, mechanisms, risk factors, prevention, and management. Expert Rev. Gastroenterol. Hepatol..

[6] Zhornitskiy A., Berry R., Han J.Y., Tabibian J.H. (2019). Hemobilia: Historical overview, clinical update, and current practices. Liver Int..

[7] Achkar J.M., Joseph G. (2012). Independent association of younger age with hemoptysis in adults with pulmonary tuberculosis. Int. J. Tuberc. Lung Dis..

[8] Ogata H., Moriwaki A., Nakagawa T., Sakoda S., Ishimatsu A., Taguchi K., Aso H., Nogami H., Kadowaki M., Tateshi Y. (2021). Association of serum antibodies against the Mycobacterium avium complex and hemoptysis: A cross-sectional study. BMC Infect. Dis..

[9] Linz C.M., Modi R.M., Krishna S.G. (2017). A dual-modality approach of endobiliary radiofrequency ablation and self-expandable metal stent placement to control malignant hemobilia. Endoscopy.

[10] Moon S.Y., Heo J., Jung M.K., Cho C.M. (2022). Biliary Self-Expandable Metal Stent Could Be Recommended as a First Treatment Modality for Immediate Refractory Post-Endoscopic Retrograde Cholangiopancreatography Bleeding. Clin. Endosc..

[11] Husnain A., Aadam A.A., Keswani R., Sinha J., Caicedo J.C., Duarte A., Stiff K., Reiland A., Cacho D.B., Salem R. (2025). Outcomes of percutaneous endobiliary radiofrequency ablation in managing resistant benign biliary strictures: A retrospective analysis. Br. J. Radiol..

[12] Özdemir M., Küçükay F., Özdemir F.A.E., Acu R., Tola M., Yurdakul M. (2018). Percutaneous endobiliary radiofrequency ablation for refractory benign hepaticojejunostomy and biliary strictures. Diagn. Interv. Imaging.

